# Assessment of the Danish DemTool intervention trial on family caregivers

**DOI:** 10.1002/alz70858_100731

**Published:** 2025-12-25

**Authors:** Emma Sofie Kjær Pedersen, T Rune Nielsen, Janet Janbek, Ann Nielsen, Dagny Ros Nicolaisdottir, Gunhild Waldemar

**Affiliations:** ^1^ Danish Dementia Research Centre, Copenhagen University Hospital ‐ Rigshospitalet, Copenhagen, Denmark; ^2^ University of Copenhagen, Copenhagen, Denmark; ^3^ Danish Dementia Research Centre, Dept. of Neurology, Copenhagen University Hospital ‐ Rigshospitalet, Copenhagen, Denmark; ^4^ Department of Clinical Medicine, University of Copenhagen, Copenhagen, Denmark

## Abstract

**Background:**

People with dementia are often in need of comprehensive support, depending on the stage of the disease, from both family members and healthcare professionals. Caring for a sick relative can be stressful and may negatively impact the caregiver's health and wellbeing. Studies show that family caregivers of people with dementia often experience stress, depression, and a reduced quality of life. A growing number of effective interventions focus on increasing the wellbeing of family caregivers of people with dementia. However, evidence‐based psychosocial interventions focusing on emotional and psychological support are sparse. The Danish Dementia Research Centre has developed ‘DemTool – supporting life with dementia’, an intervention focusing on the quality of life for people with dementia and their family caregivers. The DemTool intervention was a pragmatic multicenter controlled trial, conducted across 36 Danish municipalities. Thus, considering the sparsity of such psychosocial interventions, an assessment of the DemTool is needed to provide crucial evidence for the effect of psychosocial interventions for family caregivers of people with dementia, which is important for primary care. We aim to assess the effects of the DemTool intervention trial on health‐related quality of life, caregiver distress, caregiver experience, loneliness, and well‐being in family caregivers of people with dementia.

**Method:**

This study consists of adult family caregivers who have completed baseline and follow‐up questionnaires including demographics and validated measures of health‐related quality of life and wellbeing. We will investigate between‐group differences in outcome measures between participants in DemTool intervention, and participants receiving treatment as usual, with analysis of variance (ANOVA). In secondary analysis, comparisons will be adjusted for relevant covariates in baseline sociodemographic variables (ANCOVA), and outcome variables, such as carer burden.

**Results:**

195 adult family caregivers of people with dementia received the intervention and completed baseline and follow‐up questionnaires. 70 adult family caregivers of people with dementia revived treatment as usual and completed baseline and follow‐up questionnaires (*n* = 265). Primarily females caring for a spouse participated in the DemTool trial.

**Conclusion:**

This study will enhance our understanding and evidence for the effectiveness of psychosocial interventions focusing on emotional and psychological support for family caregivers of people with dementia.

